# PSDAAP: Provably Secure Data Authenticated Aggregation Protocols Using Identity-Based Multi-Signature in Marine WSNs

**DOI:** 10.3390/s17092117

**Published:** 2017-09-14

**Authors:** Lifei Wei, Lei Zhang, Dongmei Huang, Kai Zhang, Liang Dai, Guojian Wu

**Affiliations:** 1 College of Information Technology, Shanghai Ocean University, Shanghai 201306, China; Lfwei@shou.edu.cn (L.W.); dmhuang@shou.edu.cn (D.H.); dailiang19931020@163.com (L.D.); wuguojian19930913@163.com (G.W.); 2 Department of Computer Science and Technology, East China Normal University, Shanghai 200241, China; 52141201001@stu.ecnu.edu.cn

**Keywords:** identity-based multi-signature, provably secure, integer factorization, data authenticated aggregation, marine WSNs

## Abstract

Data authenticated aggregation is always a significant issue for wireless sensor networks (WSNs). The marine sensors are deployed far away from the security monitoring. Secure data aggregation for marine WSNs has emerged and attracted the interest of researchers and engineers. A multi-signature enables the data aggregation through one signature to authenticate various signers on the acknowledgement of a message, which is quite fit for data authenticated aggregation marine WSNs. However, most of the previous multi-signature schemes rely on the technique of bilinear pairing involving heavy computational overhead or the management of certificates, which cannot be afforded by the marine wireless sensors. Combined with the concept of identity-based cryptography, a few pairing-free identity-based multi-signature (IBMS) schemes have been designed on the basis of the integer factorization problem. In this paper, we propose two efficient IBMS schemes that can be used to construct provably secure data authenticated aggregation protocols under the cubic residue assumption, which is equal to integer factorization. We also employ two different methods to calculate a cubic root for the cubic residue number during the signer’s private key extraction. The algorithms are quite efficient compared to the previous work, especially for the algorithms of the multi-signature generation and its verification.

## 1. Introduction

**Figure 1:**
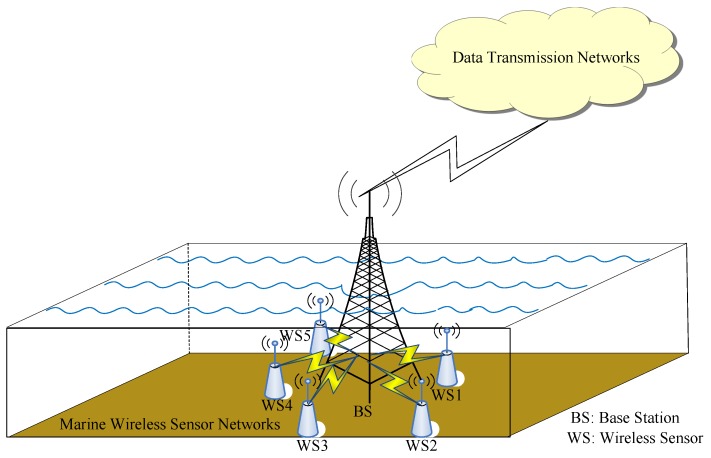
Data collection in marine wireless sensor networks (WSNs).

In most of the wireless sensor networks (WSNs), the significant issue for data collection or data aggregation always lies in the center of data transmission, both in the academia and in the industry [1,2,3]. In most scenarios of marine WSNs, all the nearby wireless sensors send their data, such as the temperature, pressure, salinity, and potential of hydrogen (pH value) in the chemistry of the environmental monitoring ocean, to a central node, which is located at a base station or a buoy for data collection, as shown in Figure 1. The central node further sends the aggregated data through the long-distance data transmission networks, such as vessel-based or satellite-based networks [4]. However, marine sensors are always deployed far away from the security monitoring. Thus, the secure data aggregation for marine sensor networks has emerged and attracted the interest of researchers and engineers. In order to mitigate the malicious attackers injecting false data, it is quite necessary for each central node to authenticate these sensing measurements from the nearby sensors in the ocean observation system [5].

Generally, a digital signature often provides the properties of authenticity and non-repudiation through checking the signed acknowledgments from senders [6]. However, in WSNs, the international standards for broadcasting authentication are very vulnerable to signature verification flooding attacks, as the excessive requests for signature verification must run out of the computational resources of those victims [7]. The scenario seems worse, as the marine wireless sensors are powered by a limited battery and cannot afford these overloaded requests in an oceanic environment. To optimize the communication and computational overhead, a variant of digital signature, named *multi-signature*, permits various signers to sign on a message individually and aggregate partial signatures to a compact signature [8].

A multi-signature can play a significant role in authenticating different sensors’ data by checking a single compact signature to cut down the communication bandwidth for marine wireless devices, as the transmission of one-bit data consumes more energy than the arithmetic operations on several bits [9]. This seems a promising way to solve the data authentication in a multi-user scenario. Since the primitive has been proposed, multi-signature schemes have been paid attention to by most of the network designers and industry engineers. However, in the past years, most of the work on multi-signature schemes has been constructed by relying on the assumed existence of *public key infrastructure* (**PKI**) [10,11]; the heavy burdens of the digital public key certificate management bring high communication overhead and storage overhead when **PKI** is applied and implemented in the wireless networks. The cases become worse when the sensors are deployed in the marine environments (denoted as **Problem 1**).

To overcome the weakness brought by **PKI**, identity-based cryptography emerges as a novel cryptographic primitive and a powerful alternative to traditional certificate-based cryptography, which has been raised early on in [12] and is further specifically designed in [13,14]. Identity-based cryptography makes some public, known information a public key, such as the device’s number, IP address, or a username, to mitigate the management problem for the public key certificates. In the extreme case that the bandwidth is a bottleneck, the identities of the signers often appear in the head of the communication packets, instead of in the transmission of the heavy public keys. Inspired by this concept, the first *identity-based multi-signature* (**IBMS**) scheme, proposed in [15], uses a mathematical technique named “bilinear mapping”, such as is used in [13], and is proved to be secure, relying on *discrete logarithm* (DL) assumptions or *computational Diffie–Hellman* (CDH) assumptions. Because the operation of bilinear mapping involves too much computational overhead [16,17], many bilinear mapping techniques are not suitable for the battery-limited sensors in marine WSNs (denoted as **Problem 2**).

As a consequence, there is great interest for cryptographic researchers to design pairing-free identity-based cryptographic schemes [18]. The first non-pairing IBMS scheme was proposed in [19] with three-round interactive communications and under R. Rivest, A. Shamir, L. Adleman (RSA) assumptions. Later, a communication efficiency-improved IBMS scheme under RSA assumptions was presented in [20] with two-round interactive communications. Yang et al. [21] proposed an efficient improved IBMS scheme that aims to save the computational resources and communication bandwidth. Even if the RSA assumption approaches the integer factorization assumptions, unfortunately, the RSA assumption has not yet been proved equal to the factorization assumption (denoted as **Problem 3**).

To satisfy the application requirements and to avoid security concerns in cryptrography, it is common practice to construct alternative cryptographic schemes under a weaker assumption—integer factorization. Recently, cryptographic researchers have been focused on finding a new construction that is proved to be secure directly on the basis of factorization. Chai [22] gave an instance of an identity-based digital signature relying on the quadratic residue assumption. Following this, Wei et al. [6] proposed IBMS schemes using quadratic residue assumptions, under weaker assumptions and a strengthened security model, achieving advantages in the computational consumption and transmission overhead. Xing [23] and Wang [24] presented identity-based signature schemes under the cubic residue assumptions. Wang proposed several signature variants relying on cubic residues, including identity-based ring signature [25], *identity-based proxy multi-signature* (**IBPMS**) [26] and threshold ring signature [27]. Wei [28] considered an *identity-based multi-proxy signature* (**IBMPS**) scheme for use in a cloud-based data authentication protocol. Zhang [29] proposed a secure multi-entity delegated authentication protocol based on an *identity-based multi-proxy multi-signature* (**IBMPMS**) for mobile cloud computing. Unfortunately, none considered constructing IBMS schemes directly based on cubic residues (denoted as **Problem 4**).

Facing the above problems, this work constructs IBMS schemes relying on the cubic residue assumption equal to integer factoring. Our schemes have merits not only in the efficiency aspect, where we do not rely on the bilinear pairing maps or over exponentiations, but also in the security aspect, where we prove them to be secure under a weaker assumption of factoring to achieve stronger security. The contributions for this paper can be summarized as follows.

1. We have proposed two efficient IBMS schemes, denoted as **IBMS^CR^−1** and **IBMS^CR^−2**, which are suitable for data aggregation among the sensors and collectors in marine WSNs.
2. We formally define the security of IBMS and prove **IBMS^CR^−1** to be secure, relying on the cubic residues in a random oracle model. The computational cost of **IBMS^CR^−1** is lower, as the exponentiations are cubic exponentials.
3. To enhance efficiency, the total computational cost of **IBMS^CR^−2** is almost four-fifths that of **IBMS^CR^−1** in implementation. We also prove the security of **IBMS^CR^−2** on the basis of the cubic residues equalling integer factoring in the random oracle model.

The organization of this paper is as follows. Section 2 gives necessary preliminaries, and Section 3 gives the formal definition of the security model. In Section 4 and Section 5, we propose two concrete IBMS schemes, **IBMS^CR^−1** and **IBMS^CR^−2**, as well as outline their correctness and full security proof. Section 6 gives the performance comparison. Section 7 gives the conclusion for the paper.

## 2. Preliminaries

Some fundamental concepts are introduced simply, for further explaining the construction and security proof.

### 2.1. Cubic Residue

We first introduce the definition of the cubic residue.

**Definition** **1** (Cubic residue [23]). For an integer N≡1(mod3), a cubic residue modulo N, $c\in Z_{N}^{*}$, if $x^{3}\equiv c\;\left(\mathrm{mod}\;N\right)$ for some $x\in Z_{N}^{*}$.

Because the module *N* is a product for unknown *p* and *q*, it is difficult to obtain *x* from a cubic residue *c*, that is, the difficulty of obtaining *x* from *c* is equal to the factorization of *N*.

### 2.2. Cubic Residue Symbol in Eisenstein Ring

Following the work in [23,30,31], we let ω denote a complex root of $z^{2}+z+1=0$, which means that ω is a cubic root of 1. We also have $\omega^{2}=-1-\omega =\bar{\omega }$, where $\bar{\omega }$ is the conjugate complex of ω. The Eisenstein ring is defined as the set Z[ω]={a+bω|a,b∈Z}. We introduce the cubic residue symbol as follows:

$${\left(\frac{\cdot }{\cdot }\right)}_{3}:Z\left[\omega \right]\times \left(Z\left[\omega \right]-\left(1-\omega \right)Z\left[\omega \right]\right)\to \left\{0,1,\omega ,\omega^{2}\right\}$$

For a prime *p* in Z[ω] where *p* is not associated to 1−ω, we have

$${\left(\frac{\alpha }{p}\right)}_{3}=\alpha^{(N(p)-1)/3}\;\;\left(\mathrm{mod}\;p\right)$$

where $N\left(p\right)=p\cdot \bar{p}$ is defined as the norm of *p*.

### 2.3. Some Useful Theorems

**Theorem** **1** (Factorization Theorem [23]). *Let N=pq, where p and q are large primes. Let c be a cubic residue modulo N, and $r_{1}$ and $r_{2}$ be c’s two cubic roots modulo N; that is, $r_{1}^{3}\equiv r_{2}^{3}\equiv c\;\left(\mathrm{mod}\;N\right)$ and $r_{1}\neq r_{2}\;\left(\mathrm{mod}\;N\right)$. N can be factored by taking $\gcd(r_{1}-r_{2},N)$ in polynomial time, where gcd(x,y) is the greatest common divisor of x and y.*

Theorem 1 is easily validated, as if $r_{1}^{3}\equiv r_{2}^{3}\equiv c\;\left(\mathrm{mod}\;N\right)$, we have $\left(r_{1}-r_{2}\right)\left(r_{1}^{2}+r_{1}r_{2}+r_{2}^{2}\right)\equiv 0\;\left(\mathrm{mod}\;N\right)$. There must exist an integer *k* such that $\left(r_{1}-r_{2}\right)\left(r_{1}^{2}+r_{1}r_{2}+r_{2}^{2}\right)=kpq$. If $r_{1}\neq r_{2}\;\left(\mathrm{mod}\;N\right)$, $r_{1}-r_{2}$ cannot be a multiple of *N* at the same time; $r_{1}-r_{2}$ must contain a non-trivial divisor of *N*, which is *p* or *q*. Therefore, the integer *N* can be factored by Theorem 1. However, the two cubic roots satisfying $r_{1}\equiv r_{2}\;\left(\mathrm{mod}\;N\right)$ cannot lead directly to factoring the integer *N*.

The following theorem shows a solution to compute a $3^{\ell }$-th root of a cubic residue without factoring *N*.

**Theorem** **2.** Let ω≡1(mod3), ℓ>0, c be a cubic residue modulo N, and $X\in Z_{N}^{*}$ satisfy

$$c^{\omega }\equiv X^{3^{\ell }}\;\;\left(\mathrm{mod}\;N\right)$$

*Then we can easily calculate the cubic root y; that is, $y^{3}\equiv c\;\left(\mathrm{mod}\;N\right)$.*

Because ω≡1(mod3), we can denote $\omega =3^{r}\left(3\delta +1\right)$; following this,

$$c^{\omega }\equiv c^{3^{r}\left(3\delta +1\right)}\equiv X^{3^{\ell }}\;\left(\mathrm{mod}\;N\right)$$

We take the $3^{r}$-th root and obtain

$$c^{3\delta +1}\equiv X^{3^{\ell }-r}\;\;\left(\mathrm{mod}\;N\right)$$

Because $c^{3\delta +1}=c^{3\delta }\cdot c$, we have

$$c\equiv \frac{X^{3^{\ell }-r}}{c^{3\delta }}\equiv {\left(\frac{X^{3^{\ell }-r-1}}{c^{\delta }}\right)}^{3}\;\left(\mathrm{mod}\;N\right).$$

Let $y=X^{3^{\ell }-r-1}/c^{\delta }$; then we have $y^{3}\equiv c\;\left(\mathrm{mod}\;N\right)$

Theorem 2 can be used in the security proof for **IBMS^CR^−1**. We introduce the following Theorem [24,29] regarding the cubic residue used in the security proof for **IBMS^CR^−2**.

**Theorem** **3**  (Cubic residue construction [24,29]). If p and q are two primes with p≡2(mod3) and q≡4 or 7(mod9), it is easy to produce a cubic residue modulo N. Let nc be a non-cubic modulo q, for any $h\in Z_{N}^{*}$; we can compute that $\eta =\frac{\left(q-1)\;\;(\mathrm{mod}\;9\right)}{3}$ , λ=η(mod2)+1, β=(q−1)/3, $\xi \equiv nc^{\eta \beta }\;\left(\mathrm{mod}\;q\right)$, $\tau \equiv h^{\lambda \beta }\;\left(\mathrm{mod}\;q\right)$ and

$$b=\left\{\begin{array}{cc} 0, & if\;\tau =1\\ 1, & if\;\tau =\xi \\ 2, & if\;\tau =\xi^{2} \end{array}\right.$$

*We can construct a cubic residue C modulo N; that is, $C=nc^{b}\cdot h\;\left(\mathrm{mod}\;N\right)$.*

**Theorem** **4.** *Let p, q, N, C, and η be defined as in Theorem 3; we can calculate a cubic root s of $C^{-1}$ by $s\equiv C^{[2^{\eta -1}\left(p-1\right)\left(q-1\right)-3]/9}\;\left(\mathrm{mod}\;N\right).$ Note that $s^{3}\cdot C\equiv 1\;\left(\mathrm{mod}\;N\right)$.*

## 3. Formal Definition and Security Model

### 3.1. Formal Definition

We assume that there exist *n* distinct signers, named $ID_{1},ID_{2},\ldots ,ID_{n}$, to authenticate a message *m* by cooperatively generating a multi-signature mσ. The signer $ID_{i}$ is denoted as $signer_{i}$.

**Theorem** **5.** *A typical IBMS scheme is always made up of six algorithms, that is, **Setup**, **Extra**, **Sign**, **Verify**, **MSign**, and **MVerify**. We describe each of them as follows.*

- **Setup**: (mpk,msk)←**Setup**(1${}^{k}$). The algorithm is controlled by the key generator center (**KGC**). The **KGC** generates the system’s master public keys mpk and master secret keys msk when it is given the security parameter k.
- ***Extra***: $sk_{ID}\leftarrow$ ***Extra** (mpk, msk, ID). The algorithm is also controlled by the **KGC**, given msk, mpk, and a user’s identity ID, such as a string. It returns the private key $sk_{ID}$ through secure channels.*
- ***Sign**: σ←**Sign** (mpk, sk, m, ID): The signer uses its private key sk, the identity ID, and the message to be signed m to generate a signature σ on m.*
- ***Verify***: {0,1}← ***Verify** (mpk, ID, m, σ): The algorithm takes the signer’s identity ID, the data m, and a candidate signature σ. If σ is a valid signature, it returns 1. Otherwise, it returns 0.*
- ***MSign***: mσ← ***MSign** (mpk, sk, m, IDSet). The signer with the private sk joins in the multi-signing algorithm, which needs additional parameters, including a message m and an identity set* $IDSet=\{ID_{1},ID_{2},\ldots ,ID_{n}\}$ *containing all the identities of the signers. After several rounds of interactive communication, **MSign** generates a multi-signature mσ*.
- ***MVerify***: {0,1}← ***MVerify** (mpk, IDSet, m, mσ). The algorithm returns 1 if mσ is a valid multi-signature on the message m by authenticating the signers in IDSet.*

***Correctness.*** When all of the participating signers honestly and correctly execute the algorithm **MSign** using the private keys, derived from the algorithm **Extra**, each of the signers will end the algorithm by obtaining a local multi-signature mσ such that

MVerify(IDSet,m,mσ,mpk)=1

where all mpk and msk are generated by the algorithm **Setup** and IDSet includes *n* identities $ID_{1},ID_{2},\ldots ,ID_{n}$ for any messages $m\in {\left\{0,1\right\}}^{*}$.

### 3.2. Security Model

This considers an extreme case: the adversary A compromising the n−1 participants and leaving *only one* honest user, denoted $signer_{1}$. The $signer_{1}$ user is controlled by the challenger C. When the game starts, C gives A the honest identity of $signer_{1}$ and allows A to compromise the other signers’ private keys. It also assume that a secure channel between the signers is not guaranteed. All of the communication among the signers can be eavesdropped upon. C provides A a hash oracle, a key extraction oracle and a multi-sign oracle. A’s final target is to successfully forge a multi-signature.

**Definition** **2.** *Considering the games between A and C.*

- ***Setup***: C *executes the algorithm to generate the master public keys mpk and sends mpk to* A.
- ***Query***: *: A is allowed to query to C in an adaptive way.*
  - ***Extraction-query** (mpk, ID). C executes **Extra** to obtain $sk_{ID}$ and sends to A when A asks for the private key of $signer_{ID}$.*
  - ***Multi-signature query** (mpk, m, IDSet) C obtains a multi-signature mσ and sends to A when A asks for the multi-signature mσ on m and IDSet.*
  - ***Hash-query**. C chooses the returned values by itself and sends to A when A asks.*
- **Forgery**. A makes a multi-signature as a forgery, that is, $m\sigma^{*}$ on $m^{*}$ for $IDSet^{*}$, which contains at least one uncompromised user’s identity; meanwhile, A never sends $\left(mpk,IDSet^{*},m^{*}\right)$ to the multi-signature query.

**Definition** **3** **(Attack** **Goals).** *The advantage $Adv_{A}^{\mathrm{IBMS}}$ in breaking the KG(k) problems is defined as*

$$\begin{array}{c} Adv_{A}^{\mathrm{IBMS}}\left(k\right)=\mathrm{Pr}\left[x^{3^{\ell }}\equiv y\;\left(\mathrm{mod}\;N\right)\left|\begin{array}{c} \left(N,p,q)\leftarrow \mathrm{KG}(k\right)\\ y\leftarrow Z_{N}^{*}\\ x\leftarrow A(N,\ell ,y) \end{array}\right.\right] \end{array}$$

**Definition** **4** **(Unforgeability).** *An adversary A$\left(t,q_{H},q_{E},q_{S},n,\epsilon \right)$ breaks the scheme if A executes for a time of t at most, and makes at most $q_{H}$ hash queries, $q_{E}$ extraction queries, and $q_{S}$ multi-signature queries with n participants, and $Adv_{A}$ is at least ϵ. An IBMS scheme $\left(t,q_{E},q_{S},q_{H},n,\epsilon \right)$ has unforgeability if there exists no attacker A$\left(t,q_{H},q_{E},q_{S},n,\epsilon \right)$ that breaks it.*

## 4. Concrete Construction of **IBMS^CR^-1**

### 4.1. Construction

Inspired by the previous work [6,22,23], we propose a concrete identity-based multi-signature scheme (**IBMS^CR^−1**) with three-round interactive communications among the marine sensors and the generation of a single multi-signature as an authenticated tag.

- Setup (k,ℓ): The key generator center inputs security parameters *k* and *ℓ*, and then:
  1. Chooses two random primes *p* and *q*, such that p≡q≡1(mod3) and (p−1)(q−1)/9≡−1(mod3). Without loss of generality, we assume that (p−1)/3≡−1(mod3), (q−1)/3≡1(mod3).
  2. Chooses two random primes $\pi_{1}$ and $\pi_{2}$ from the Eisenstein ring Z[ω], *s.t.* the norms satisfy $N(\pi_{1})=p$ and $N(\pi_{2})=q$.
  3. Computes N=p∗q. We let $A+B\omega =\pi_{1}\pi_{2}$, A,B∈Z, and then compute $C=-AB^{-1}\;\left(\mathrm{mod}\;N\right)$. Note that ${\left(\frac{C}{p}\right)}_{3}=\omega^{2}$, and ${\left(\frac{C}{q}\right)}_{3}=\omega$.
  4. Chooses a random number $a\in Z_{N}^{*}$ such that ${\left(\frac{a}{N}\right)}_{3}=\omega$.
  5. Computes $d=\frac{1}{3}\left[\frac{1}{9}\left(p-1\right)\left(q-1\right)+1\right]$.
  6. Selects three hash functions $h_{1}\left(\cdot \right)$, $h_{2}\left(\cdot \right)$, and $h_{3}\left(\cdot \right)$ such that $h_{1}\left(\cdot \right):{\left\{0,1\right\}}^{*}\to Z_{N}^{*}$, $h_{2}$ and $h_{3}\left(\cdot \right):{\left\{0,1\right\}}^{*}\to {\left\{0,1\right\}}^{\ell }$.

As a result of the step **Setup**, the master secret key is msk=(p,q,d), which is securely stored, and the public parameter is $mpk=(N,h_{1},h_{2},h_{3},a,C,\ell )$.

- Extra (*mpk, msk, ID*): **KGC** inputs the identity ID, computes the hash value of ID as $h_{1}\left(ID\right)$ and obtains a first symbol $c_{ID,1}$ such that
  $$c_{ID,1}=\left\{\begin{array}{cc} 0, & \;\mathrm{if}\;\;{\left(\frac{h_{1}\left(ID\right)}{N}\right)}_{3}=1\\ 1, & \;\mathrm{if}\;\;{\left(\frac{h_{1}\left(ID\right)}{N}\right)}_{3}=\omega^{2}\\ 2, & \;\mathrm{if}\;\;{\left(\frac{h_{1}\left(ID\right)}{N}\right)}_{3}=\omega \end{array}\right.$$
  We let $h=a^{c_{ID,1}}\cdot h_{1}\left(ID\right)$ and we have ${\left(\frac{h}{N}\right)}_{3}=1$. Following this, **KGC** computes a second symbol $c_{ID,2}$ such that
  $$c_{ID,2}=\left\{\begin{array}{cc} 0, & \;\mathrm{if}\;\;{\left(\frac{h}{p}\right)}_{3}={\left(\frac{h}{q}\right)}_{3}=1\\ 1, & \;\mathrm{if}\;\;{\left(\frac{h}{p}\right)}_{3}=\omega ,{\left(\frac{h}{q}\right)}_{3}=\omega^{2}\\ 2, & \;\mathrm{if}\;\;{\left(\frac{h}{p}\right)}_{3}=\omega^{2},{\left(\frac{h}{q}\right)}_{3}=\omega \end{array}\right.$$
  We let $I_{ID}=C^{c_{ID,2}}\cdot a^{c_{ID,1}}\cdot h_{1}\left(ID\right)$. It is easy to find that $I_{ID}\in {\mathrm{CR}}_{N}$, as ${\left(\frac{I_{ID}}{p}\right)}_{3}={\left(\frac{I_{ID}}{q}\right)}_{3}=1.$ Finally, **KGC** extracts the private key $sk_{ID}$ as a $3^{\ell }$-th root of $I_{ID}$:
  (1) $$sk_{ID}\equiv I_{ID}^{d^{\ell }}\;\;\left(\mathrm{mod}\;N\right)$$
  **KGC** sends $sk_{ID}$ as well as ($c_{ID,1},c_{ID,2}$) to signer ${}_{ID}$ secretly. Note that $I_{ID}\equiv sk_{ID}^{3^{\ell }}\;\left(\mathrm{mod}\;N\right)$. Following this, we denote $\tilde{ID}=\left\{ID,c_{ID,1},c_{ID,2}\right\}$.
- Sign and verify: These two algorithms can be derived from [23].
- MSign $\left(mpk,sk_{1},m,IDSet\right)$: For simplicity, **IBMS^CR^−1** is described from the $MS_{1}$’s point of view. Given the $MS_{1}$’s private key $sk_{1}$, the message *m* and the identity set $IDSet=\{\tilde{ID_{1}},\tilde{ID_{2}},\ldots ,\tilde{ID_{n}}\}$, $MS_{1}$ executes the following algorithm from Algorithm 1. **MSign** generates mσ=(w,u) as the multi-signature.
- MVerify (*mpk, IDSet, m, mσ*). The algorithm verifies by the following three steps.
  (1) For i=1,2,…,n, it computes $I_{i}\equiv C^{c_{ID_{i},2}}\cdot a^{c_{ID_{i},1}}\cdot h_{1}\left(ID_{i}\right)\;\left(\mathrm{mod}\;N\right)$.
  (2) It computes $\hat{R}\equiv u^{3^{\ell }}{\left(\prod_{i=1}^{n}I_{i}\right)}^{-w}\;\left(\mathrm{mod}\;N\right)$.
  (3) It checks whether
    (2) $$w=h_{3}(\hat{R}∥IDSet∥m)$$
    is satisfied. If Equation (2) is satisfied, **MVerify** returns 1. This means mσ is valid. Otherwise **MVerify** returns 0.

### 4.2. Correctness

The correctness follows:

$$u^{3^{\ell }}\equiv \prod_{i=1}^{n}u_{i}^{3^{\ell }}\equiv \prod_{i=1}^{n}r_{i}^{3^{\ell }}sk_{i}^{w3^{\ell }}\equiv \prod_{i=1}^{n}R_{i}I_{i}^{{\left(3d\right)}^{\ell }w}\equiv R\prod_{i=1}^{n}I_{i}^{w}\;\left(\mathrm{mod}\;N\right)$$

We have $\hat{R}\equiv R\equiv u^{3^{\ell }}\prod_{i=1}^{n}I_{i}^{-w}\;\left(\mathrm{mod}\;N\right)$.

**Algorithm 1:**
**The MSign Algorithm in IBMS ^CR^−1**. **Input**: the master public key mpk, the private key sk, the identity set IDSet, the message to be signed *m*; **Output**: a multi-signature mσ.  1. Each $MS_{i}$ randomly selects $r_{i}\in Z_{N}^{*}$ and computes $R_{i}\equiv r_{i}^{3^{\ell }}\;\left(\mathrm{mod}\;N\right)$ and $t_{i}=h_{2}\left(R_{i}\right)$.  2. $MS_{i}$ only broadcasts $t_{i}$ to other signers $MS_{j}$ (j≠i) in IDSet and keeps $R_{i}$ temporarily.  3. After receiving $t_{i}$ from $MS_{i}$ (2≤i≤n), $MS_{1}$ then broadcasts $R_{1}$ to other $MS_{i}$.  4. After receiving $R_{i}$ from $MR_{i}$, $MS_{1}$ checks whether $t_{i}=h_{2}\left(R_{i}\right)$ for 2≤i≤n is satisfied.  5. If one of these fails, the algorithm stops, which means the attackers have mixed invalid partial signatures. Otherwise, $MS_{1}$ sets $R\equiv \prod_{i=1}^{n}R_{i}\;\left(\mathrm{mod}\;N\right)$, $w=h_{3}\left(R∥IDSet∥m\right)$, and $u_{1}\equiv r_{1}\cdot sk_{1}^{w}\;\left(\mathrm{mod}\;N\right)$.  6. $MS_{1}$ broadcasts $u_{1}$ to other $MS_{i}$.  7. After receiving $u_{i}$ from $MS_{i}$, $MS_{1}$ aggregates these by $u\equiv \prod_{i=1}^{n}u_{i}\;\left(\mathrm{mod}\;N\right)$.  8. Each $MS_{i}$ locally generates a multi-signature mσ=(w,u). **Return**
mσ;

### 4.3. Security Proof

**IBMS^CR^−1** is provably secure under the factorization in the random oracle model.

**Theorem** **6.** *If the factorization problem is $\left(t^{\prime },\epsilon^{\prime }\right)$-hard, **IBMS^CR^−1** is $\left(t,q_{E},q_{H},q_{S},n,\epsilon \right)$-secure against existential forgery attackers under the adaptively chosen message attack and chosen identity attack. We have estimates for $t^{\prime }$ and $\epsilon^{\prime }$ as follows:*

(3) $$\epsilon^{\prime }⩾\frac{2\epsilon^{2}}{3(q_{H}+1)}-\left(\frac{2nq_{S}q_{H}+n^{2}q_{S}^{2}+q_{H}^{2}}{2^{\ell_{R}}\cdot \left(q_{H}+1\right)}+\frac{nq_{S}}{2^{\ell_{0}-1}}\right)\epsilon -\frac{1}{3\cdot 2^{\ell -1}}$$

**Proof.**  We assume C is given a factorization instance *N* for a product of unknown *p* and *q*, and obtain the result of *p* or *q* with a non-negligible probability. C plays with A as follows.

Firstly, C selects $a\in Z_{N}^{*}$, such as a non-cubic residue and a secure parameter ℓ⩾160 (the length of *ℓ* has been discussed and suggested in [22]), and sends (N,a,ℓ) to A as mpk. C manages several lists: one signature list and three hash lists.

Then, C starts to answer according to A’s queries, as follows.

- $h_{1}$**-Query** (ID): C manages a list $\left(ID,h_{1},s,c_{ID,1},c_{ID,2}\right)$. When A requests the identity ID, C answers as $h_{1}$. $\left(c_{ID,1},c_{ID,2}\right)\in {\left\{0,1\right\}}^{2}$ in two bits and $s\in Z_{N}^{*}$ is used as a secret key. When A asks on ID, C answers $h_{1}$ if ID has existed in the $h_{1}$-list. Otherwise, C randomly selects $s\in Z_{N}^{*}$ and $\left(c_{ID,1},c_{ID,2}\right)\in {\left\{0,1\right\}}^{2}$, calculates
  (4) $$h_{1}\equiv \frac{s^{3^{\ell }}}{{\left(-1\right)}^{c_{ID,2}}\cdot {\left(a\right)}^{c_{ID,1}}}\;\left(\mathrm{mod}\;N\right)$$
  and returns the answer $h_{1}$ to A, adding $\left(ID,h_{1},s,c_{ID,1},c_{ID,2}\right)$ to the $h_{1}$-list.
- $h_{2}$**-Query** (R): C manages a list $\left(R,h_{2}\right)$. When A asks on *R*, C answers $h_{2}$ if *R* has existed in the $h_{2}$-list. Otherwise, C randomly selects $h_{2}\in {\left\{0,1\right\}}^{\ell_{0}}$, adds $\left(R,h_{2}\right)$ into the $h_{2}$-list and returns $h_{2}$.
- $h_{3}$**-Query** (R,m,IDSet): C manages a list $\left(R,m,IDSet,h_{3}\right)$. When A asks on (R,m,IDSet), C returns $h_{3}$ if (R,m,IDSet) has existed in the $h_{3}$-list. Otherwise, C randomly selects $h_{3}\in Z_{N}^{*}$, returns $h_{3}$, and adds $\left(R,m,IDSet,h_{3}\right)$ to the $h_{3}$-list.
- **Extraction query** (ID): C executes an additional $h_{1}$-query if ID does not yet exist in the $h_{1}$-list and returns *s* and $\left(c_{ID,1},c_{ID,2}\right)$.
- **Multi-signature queries**: C checks in the $h_{1}$-list for whether $ID_{1}$ exists. If $ID_{1}$ is already in the $h_{1}$-list, C has obtained the private key of $signer_{1}$ and simulates the game as the real algorithm **MSign** $\left(sk_{1},IDSet,m\right)$ using the secret key $sk_{1}=s_{1}$. Otherwise, C does not have the private key of $signer_{1}$ and executes the following steps:
  - C plays as $signer_{1}$, and randomly chooses $t_{1}\leftarrow {\left\{0,1\right\}}^{\ell_{0}}$, broadcasting $t_{1}$ to other signers. C also waits to receive $t_{2},t_{3},\cdots ,t_{n}$ from others; it randomly selects $w\leftarrow {\left\{0,1\right\}}^{\ell }$ and $u_{1}\leftarrow Z_{N}^{*}$, and calculates
    (5) $$R_{1}=u_{1}^{3^{\ell }}{\left({\left(-1\right)}^{c_{ID_{1},2}}\cdot a^{c_{ID_{1},1}}\cdot h_{1}\left(ID_{1}\right)\right)}^{-w}$$
    If $R_{1}$ already exists in the $h_{2}$-list, C stops. Otherwise, C sets $\left(R_{1},t_{1}\right)$ in the $h_{2}$-list. C looks up $R_{i}$ such that $\left(R_{i},t_{i}\right)$ where 2⩽i⩽n. If for some *i* the record is found, C also stops. Otherwise, C calculates $R=\prod_{i=1}^{n}R_{i}\;\left(\mathrm{mod}\;N\right)$ and sets $h_{3}\left(R∥S∥m\right)=w$, or stops if the entry has already existed.
  - C sends $R_{1}$ to other signers. After receiving $R_{2}^{\prime },\cdots ,R_{n}^{\prime }$ from the signers, C verifies that $h_{2}\left(R_{i}^{\prime }\right)\overset{?}{=}t_{i}$. C ends up with the protocol if one of these does not satisfy this, which means A has to guess the results of the hash value. If $R_{i}\neq R_{i}^{\prime }$ for some *i*, C stops. C sends $u_{i}$ to the signers, receives $u_{2},u_{3},\cdots ,u_{n}$, and calculates $u=\prod_{i=1}^{n}u_{i}\;\left(\mathrm{mod}\;N\right)$. Finally, C sends mσ=(w,u) to A.

At the end of the game, A generates a multi-signature $m\sigma^{*}=\left(w^{*},u^{*}\right)$ on message $m^{*}$. C calculates

(6) $$\begin{array}{c} R^{*}\leftarrow {\left(u^{*}\right)}^{3^{\ell }}\prod_{i=1}^{n}{\left({\left(-1\right)}^{c_{ID_{i}^{*},2}}a^{c_{ID_{i}^{*},1}}h_{1}\left(ID_{i}^{*}\right)\right)}^{-w^{*}} \end{array}$$

and makes an additional query $h_{3}(R^{*}∥IDSet^{*}∥m^{*})$. We let $U\subseteq IDSet^{*}=\left\{ID_{1}^{*},ID_{2}^{*},\ldots ,ID_{n}^{*}\right\}$ denote the honest IDSet, that is, A never compromised. If A succeeded in forgery, that is,

- **MVerify** $(mpk,IDSet^{*},m^{*},\sigma^{*})=1$
- U≠∅
- A has never queried $\left(IDSet^{*},m^{*}\right)$ to the signature oracle then C checks the $h_{1}$-list. If the multi-signature is valid, we can obtain
  (7) $$\begin{array}{ccc} u{{}^{*}}^{3^{\ell }} & \equiv & R^{*}\prod_{i=1}^{n}{\left({\left(-1\right)}^{c_{ID_{i},2}}a^{c_{ID_{i},1}}h_{1}\left(ID_{i}^{*}\right)\right)}^{w^{*}}\\ & \equiv & R^{*}\prod_{i=1}^{n}{s_{i}^{*}}^{3^{\ell }w^{*}}\;\left(\mathrm{mod}\;N\right) \end{array}$$

We let $s^{*}\leftarrow \prod_{i=1}^{n}{\left(s_{i}^{*}\right)}^{3^{\ell }}\;\left(\mathrm{mod}\;N\right)$ and produce $\left(s^{*},\sigma^{*}\right)$.

To factor *N* by applying the rewinding technique, C plays with A once again using the random tapes, which are the same as for the first time. Because C previously recorded the transcripts, C obtains the same results for A’s queries.

When A queries for $h_{3}$, C randomly selects an alternative answer $w^{\prime }$ instead of *w*, as, in the second run, the $h_{1}$- and $h_{2}$-query are equal to those of the first round.

C generates (s,mσ) and $\left(s^{\prime },m\sigma^{\prime }\right)$ such that

$$u^{3^{\ell }}\equiv Rs^{w}\;\;and\;\;u^{\prime 3^{\ell }}\equiv R^{\prime }s^{\prime w^{\prime }}$$

By $R=R^{\prime }$, $m=m^{\prime }$ and $s=s^{\prime }$, we have

(8) $$\begin{array}{c} {\left(\frac{u}{u^{\prime }}\right)}^{3^{\ell }}\equiv s^{\left(w-w^{\prime }\right)}\;\left(\mathrm{mod}\;N\right) \end{array}$$

Because $w\neq w^{\prime }\in {\left\{0,1\right\}}^{\ell_{0}}$ and $\ell_{0}<\ell$, we can obtain $|w-w^{\prime }|<3^{\ell }$. According to Theorem 2, C can calculate a cubic root $\tilde{s}$ where ${\tilde{s}}^{3}=s$. Meanwhile, C checks the $h_{1}$-list to search for an entry in which $ID_{i}\in IDSet$ and calculates $\bar{s}=\prod_{i\in IDSet}s_{i}^{3^{\ell -1}}$.

Therefore, ${\tilde{s}}^{3}\equiv {\bar{s}}^{3}\equiv s\;\left(\mathrm{mod}\;N\right)$. If $\bar{s}\neq \tilde{s}\;\left(\mathrm{mod}\;N\right)$, *N* can be factored by Theorem 1. Otherwise, C cannot factor *N*. The probability that $\tilde{s}\neq \bar{s}\;\left(\mathrm{mod}\;N\right)$ is 2/3.

Finally, we calculate the probability that C returns a *valid* result. Because most of the simulation game is similar to in [6], we set $\epsilon^{\prime }$, ϵ and $\epsilon^{*}$ as the probability to factor *N* by C, the probability to forge a multi-signature in practice by A and the probability to succeed in the first run before the rewinding technique by A, respectively.

We have

(9) $$\epsilon^{*}⩾\epsilon -\frac{q_{S}\left(q_{H}+nq_{S}\right)}{2^{\ell_{N}}}-\frac{{\left(q_{H}+nq_{S}\right)}^{2}}{2^{\ell_{N}+1}}-\frac{2q_{S}\left(q_{H}+q_{S}\right)}{2^{\ell_{N}}}-\frac{nq_{S}}{2^{\ell_{0}}}$$

Furthermore, according to the forking lemma [32], we can easily obtain

(10) $$frk⩾\epsilon^{*}\left(\frac{\epsilon^{*}}{q_{H}}-\frac{1}{2^{\ell }}\right)⩾\frac{{\epsilon^{*}}^{2}}{q_{H}+1}-\frac{1}{2^{\ell }}$$

The probability that C succeeds to factor *N* is

(11) $$\begin{array}{ccc} \epsilon^{\prime } & ⩾ & \frac{2}{3}\cdot frk⩾\frac{2{\epsilon^{*}}^{2}}{3(q_{H}+1)}-\frac{1}{3\cdot 2^{\ell -1}}\\ & ⩾ & \frac{2\epsilon^{2}}{3(q_{H}+1)}-\left(\frac{2nq_{S}q_{H}+n^{2}q_{S}^{2}+q_{H}^{2}}{2^{\ell_{R}}\cdot \left(q_{H}+1\right)}+\frac{nq_{S}}{2^{\ell_{0}-1}}\right)\epsilon -\frac{1}{3\cdot 2^{\ell -1}} \end{array}$$

☐

## 5. Concrete Construction of **IBMS^CR^−2**

Inspired by the related work [24,26,29], we give a more efficient IBMS construction (named **IBMS^CR^−2**), whose computational overhead in **MSign** and **MVerify** is much lower than for those in **IBMS^CR^−1**.

### 5.1. Construction

- **Setup** (k,ℓ): Given the security parameters, **Setup** can be executed as follows.
  (1) **KGC** chooses random primes *p* and *q* where p≡2(mod3) and q≡4 or 7(mod9), and calculates the product N=p·q.
  (2) A non-cubic residue *a* is selected such that $\left(\frac{a}{q}\right)=-1$.
  (3) Several computational parameters are computed:
    $$\begin{array}{ccc} \eta & = & [q-1\;(\mathrm{mod}\;9)]/3\\ \lambda & = & \eta \;(\mathrm{mod}\;2)+1\\ \beta & = & (q-1)/3\\ \xi & = & a^{\eta \beta }\;\left(\mathrm{mod}\;q\right) \end{array}$$
  (4) Three hash functions $h_{1},h_{2}$ and $h_{3}$ are picked up, where $h_{1}$:${\left\{0,1\right\}}^{*}\to Z_{N}^{*}$, $h_{2},h_{3}$:${\left\{0,1\right\}}^{*}\to {\left\{0,1\right\}}^{\ell }$.

Finally, the algorithm **Setup** outputs msk=(p,q,β) and $mpk=(N,h_{1},h_{2},h_{3},a,\eta ,\lambda )$. **KGC** keeps msk secretly.

- **Extra** (mpk,msk,ID): **KGC** computes sk as follows:
  (1) **KGC** computes $\omega =h_{1}{\left(ID\right)}^{\lambda \beta }\;\left(\mathrm{mod}\;q\right)$ and set sa symbol $c_{ID}$ according to ω and ξ:
    $$c_{ID}=\left\{\begin{array}{cc} 0, & \mathrm{if}\;\omega =1\\ 1, & \mathrm{if}\;\omega =\xi \\ 2, & \mathrm{if}\;\omega =\xi^{2} \end{array}\right.$$
    **KGC** denotes $I=a^{c_{ID}}\cdot h_{1}\left(ID\right)\;\left(\mathrm{mod}\;N\right)$.
  (2) **KGC** calculates
    (12) $$sk=I^{\frac{2^{\eta }\left(p-1\right)\left(q-1\right)-3}{9}}\;\left(\mathrm{mod}\;N\right)$$
    and securely distributes sk to the signer. We have $sk_{i}^{3}\cdot I_{i}\equiv 1\;\left(\mathrm{mod}\;N\right)$. Following this, we denote the identity by $\tilde{ID_{i}}=\left\{ID_{i},c_{ID_{i}}\right\}$.
- **Sign** and **verify**: These two algorithms can be derived from [29].
- **MSign** $\left(mpk,sk_{1},m,IDSet\right)$: Given the $MS_{1}$’s private key $sk_{1}$, the message *m* and the identity set $IDSet=\{\tilde{ID_{1}},\tilde{ID_{2}},...,\tilde{ID_{n}}\}$, $MS_{1}$ executes the following algorithm in Algorithm 2. **MSign** generates the multi-signature mσ=(w,u).
- **MVerify** (mpk,IDSet,m,mσ). The algorithm verifies by the following three steps:
  (1) For i=1,2,...,n, it computes $I_{i}=a^{c_{ID_{i}}}\cdot h_{1}\left(ID_{i}\right)$.
  (2) It computes $\hat{R}=u^{3}\cdot {\left(\prod_{i=1}^{n}I_{i}\right)}^{w}\;\left(mod\;N\right)$.
  (3) It checks whether
    (13) $$w=h_{3}(\hat{R}∥IDSet∥m)$$
    is satisfied. If Equation (13) is satisfied, **MVerify** returns 1. This means mσ is valid. Otherwise **MVerify** returns 0.

**Algorithm 2:** The MSign algorithm in **IBMS^CR^−2**. **Input**: the master public key mpk, the private key sk, the identity set IDSet, the message to be signed *m*; **Output**: a multi-signature mσ.  1. Each $MS_{i}$ randomly selects $r_{i}\in Z_{N}^{*}$ and calculates $R_{i}=r_{i}^{3}\;\left(mod\;N\right)$ and $t_{i}=h_{2}\left(R_{i}\right)$.  2. Each $MS_{i}$ broadcasts $t_{i}$ to co-signers $MS_{j}$ (j≠i).  3. After obtaining $t_{i}$ from $MS_{i}$, $MS_{1}$ broadcasts $R_{1}$ to other $MS_{i}$.  4. After receiving $R_{i}$ from other signers, $MS_{1}$ checks whether $t_{i}=h_{2}\left(R_{i}\right)$ for 2⩽i⩽n is satisfied.  5. If one of these fails, the algorithm stops, which means the attackers have mixed invalid partial signatures. Otherwise, $MS_{1}$ sets $R=\prod_{i=1}^{n}R_{i}\;\left(mod\;N\right)$, $w=h_{3}\left(R∥IDSet∥m\right)$, and $u_{1}=r_{1}\cdot sk_{1}^{w}\;\left(mod\;N\right)$.  6. $S_{1}$ broadcasts $u_{1}$ to other $MS_{i}$.  7. After receiving $u_{i}$ from $MS_{i}$, $MS_{1}$ aggregates these by $u=\prod_{i=1}^{n}u_{i}\;\left(mod\;N\right)$.  8. Each $MS_{i}$ locally generates a multi-signature mσ=(w,u). **Return**
mσ;

### 5.2. Correctness

The correctness is as follows:

$$u^{3}\cdot \prod_{i=1}^{n}I_{i}^{w}\equiv \prod_{i=1}^{n}u_{i}^{3}I_{i}^{w}\equiv \prod_{i=1}^{n}r_{i}^{3}\cdot {\left(sk_{i}^{3}\cdot I_{i}\right)}^{w}\equiv \prod_{i=1}^{n}R_{i}\equiv R\;\left(\mathrm{mod}\;N\right)$$

### 5.3. Security Proof

**IBMS^CR^−2** is secure under the factorization in the random oracle model.

**Theorem** **7.** *If integer factorization is $\left(t^{\prime },\epsilon^{\prime }\right)$-hard, our **IBMS^CR^−2** scheme is $\left(t,q_{H},q_{E},q_{S},n,\epsilon \right)$-secure against existential forgery in the random oracle model.*

Because most of the simulation game between A and C is the same, we give the security proof simply.

**Proof.**  When it is given an integer factorization instance *N*, C returns *p* or *q* if A succeeds in forging a multi-signature.

C sends $mpk=\{N,h_{1},h_{2},h_{3},a,\eta ,\lambda \}$ to A. C maintains several lists ($list_{h_{1}}$, $list_{h_{2}}$, $list_{h_{3}}$, $list_{S}$).

- $h_{1}$**-Query**. C manages a list $\left(ID,c,h_{1},s\right)$. C sends $h_{1}$ to A if ID exists when A queries the hash value of ID. Otherwise, C randomly selects $s\in Z_{N}^{*}$ and c∈{0,1,2}, sets $h_{1}\equiv s^{3}/a^{c}\;\left(\mathrm{mod}\;N\right)$, returns $h_{1}$, and adds $\left(ID,c,h_{1},s\right)$ to $list_{h_{1}}$.
- The $h_{2}$**-query**, $h_{3}$**-query** and **extraction query** are similar to **IBMS^CR^−1**.
- The **multi-signature query** is similar to **IBMS^CR^−1**, except that Equation (5) changes to
  (14) $$R_{1}=u_{1}^{3}\prod_{i=1}^{n}{\left(a^{c_{ID_{i}}}\cdot h_{1}\left(ID_{1}\right)\right)}^{-w^{*}}$$

At the end of the game, A forges $m\sigma^{*}=\left(w^{*},u^{*}\right)$ with $IDSet^{*}$ on $m^{*}$. C calculates

(15) $$R^{*}\leftarrow {\left(u^{*}\right)}^{3}\prod_{i=1}^{n}{\left(a^{c_{ID_{i}^{*}}}\cdot h_{1}\left(ID_{i}^{*}\right)\right)}^{-w^{*}}$$

and queries $h_{3}(R^{*}∥IDSet^{*}∥m^{*})$ to the hash oracle. If the forgery is valid, we obtain that

(16) $$u{{}^{*}}^{3}\equiv R^{*}\prod_{i=1}^{n}{\left(a^{c_{ID_{i}^{*}}}\cdot h_{1}\left(ID_{i}^{*}\right)\right)}^{w^{*}}\equiv R^{*}\prod_{i=1}^{n}{\left({s_{i}^{*}}^{3}\right)}^{w^{*}}\equiv R^{*}{s^{*}}^{w^{*}}\;\left(\mathrm{mod}\;N\right)$$

because $s^{*}\leftarrow \prod_{i=1}^{n}{\left(s_{i}^{*}\right)}^{3}\;\left(\mathrm{mod}\;N\right)$. C returns $\left(s^{*},w^{*},u^{*}\right)$.

We also apply the rewinding technique to factor *N*. At last, C obtains (s,w,u) and $\left(s^{\prime },w^{\prime },u^{\prime }\right)$ such that

(17) $$u^{3}\equiv Rs^{w}\;\;and\;\;u^{\prime 3}\equiv R^{\prime }s^{\prime w^{\prime }}$$

Because $R=R^{\prime }$, $m=m^{\prime }$, and $s=s^{\prime }$, we have

(18) $$\begin{array}{c} {\left(\frac{u}{u^{\prime }}\right)}^{3}\equiv s^{\left(w-w^{\prime }\right)}\;\left(\mathrm{mod}\;N\right) \end{array}$$

Because $w\neq w^{\prime }$, two cases emerge:

- If $w-w^{\prime }\equiv 1\;\left(\mathrm{mod}\;3\right)$, we denote $w-w^{\prime }=3k+1$ for an integer *k*. Therefore, $s\equiv {\left(\frac{u}{u^{\prime }\cdot s^{k}}\right)}^{3}$, that is, $\tilde{s}=\frac{u}{u^{\prime }\cdot s^{k}}$ satisfies ${\tilde{s}}^{3}\equiv s\;\left(\mathrm{mod}\;N\right)$.
- If $w-w^{\prime }\equiv -1\;\left(\mathrm{mod}\;3\right)$, we denote $w-w^{\prime }=3k-1$ for an integer *k*. Therefore, $s\equiv {\left(\frac{u\cdot s^{k}}{u^{\prime }}\right)}^{3}$, that is, $\tilde{s}=\frac{u\cdot s^{k}}{u^{\prime }}$ satisfies ${\tilde{s}}^{3}\equiv s\;\left(\mathrm{mod}\;N\right)$.

From the discussion above, C calculates a cubic root $\tilde{s}$ where ${\tilde{s}}^{3}=s$. Meanwhile C searches the entries in the $h_{1}$-list where $ID_{i}\in IDSet$ and calculates $\bar{s}=\prod_{i\in IDSet}s_{i}^{3}$. Therefore, we have ${\tilde{s}}^{3}\equiv {\bar{s}}^{3}\equiv s\;\left(\mathrm{mod}\;N\right)$. If $\bar{s}\neq \tilde{s}\;\left(\mathrm{mod}\;N\right)$, we can factor *N* by Theorem 1 with a probability that $\tilde{s}\neq \bar{s}\;\left(\mathrm{mod}\;N\right)$ of 2/3.

Thus, we have finished the proof. ☐

## 6. Performance Comparisons

The comparison of security assumptions for related works are given in Table 1. These schemes are provably secure on the basis of different hardness assumptions (such as CDH, DL, RSA, quadratic residues, and cubic residues). The aim of these schemes is to find new constructions under simpler hardness assumptions.

We denote $M_{p}$, $H_{m}$, $O_{p}$ and $E_{n}$ as the operation of scalar multiplication, map-to-point hash function, bilinear pairing, and modular exponentiation, respectively. We ran each of the above operations in a personal computer and used their times from [33] to calculate the total computational cost in the running time (milliseconds), as shown in the columns of Table 2.

We have also compared related works on the basis of the cubic residues for the computational performance evaluation in Table 3. For consistency, we used the modular exponentiation times to evaluate the **Sign** and **Verify** algorithms.

## 7. Conclusions

Data authenticated aggregation is always a significant issue for marine WSNs. Most data authenticated aggregation is based on the multi-signature, which relies on the technique of bilinear pairing involving heavy computational overhead or the management of certificates beyond marine wireless sensors. We have constructed two efficient IBMS schemes (**IBMS^CR^−1** and **IBMS^CR^−2**) based on cubic residues, which are much more suitable for data authenticated aggregation in marine WSNs. Without employing the heavy overload of a bilinear pairing technique, our schemes have been designed efficiently. Our schemes have been proven to be secure under chosen identity attacks and chosen message attacks, relying only on the hardness of the integer factorization assumptions. 

## Figures and Tables

**Table 1:** The comparison of related work on the security assumptions.

Schemes	The Underlying Mathematical Assumptions
[15]	Computational Diffie-Hellman (CDH)
[19]	Discrete Logarithm (DL)
[20]	RSA
[6]	Quadratic Residues
**IBMS^CR^-1**	Cubic Residues
**IBMS^CR^-2**	Cubic Residues

**Table 2:** The comparison of related work of IBMS on the computational performance.

Schemes	Extract	Sign	Verify	Total Time	Length
[15]	2$H_{m}$ + 2$M_{p}$	1$H_{m}$ + 4$M_{p}$	3$O_{p}$	107.52	2 |g|
[19]	1$E_{n}$	2$E_{n}$	2$E_{n}$	26.55	ℓ+|N|
[20]	1$E_{n}$	2$E_{n}$	2$E_{n}$	26.55	ℓ+2|N|
[6]	1$E_{n}$	2$E_{n}$	2$E_{n}$	26.55	ℓ+|N|
**IBMS^CR^-1**	1$E_{n}$	2$E_{n}$	2$E_{n}$	26.55	ℓ+|N|
**IBMS^CR^-2**	2$E_{n}$	1$E_{n}$	1$E_{n}$	21.24	ℓ+|N|

**Table 3:** The comparison of related work on computational performance based on the cubic residues.

Schemes	Underlying Cryptographic Primitive	Sign	Verify	Total Time
[28]	IBMPS	3$E_{n}$	3$E_{n}$	6$E_{n}$
[26]	IBPMS	1$E_{n}$	3$E_{n}$	4$E_{n}$
[29]	IBMPMS	3$E_{n}$	3$E_{n}$	6$E_{n}$
**IBMS^CR^-1**	IBMS	2$E_{n}$	2$E_{n}$	4$E_{n}$
**IBMS^CR^-2**	IBMS	1$E_{n}$	1$E_{n}$	2$E_{n}$
